# Supplementary material for: Learning curve and surgical outcome of robotic assisted colorectal surgery with ERAS program
Source: Sci Rep. 2022 Nov 29;12:20566. doi: 10.1038/s41598-022-24665-w (PMC9709162; doi:10.1038/s41598-022-24665-w)
Supplement: Supplementary file 1 — Supplementary Information 1. [file 41598_2022_24665_MOESM1_ESM.zip › LSC Raw data-Ñ[▒K/075/N_ñw╝╨░O▒Kñσ.pdf]

W52 / W52

臺中榮民總醫院

[redacted]

基本資料

- 室別： 開刀房-14室，手術科別： CRS，手術類別： 常規，日期： 2020/03/26
- 進入時間： 1140 ； 麻醉開始時間： 1145 ； Time Out時間： 1250 ； 劃刀時間： 1251 ； 手術結束時間： 1725 送出時間： 1750 ；
- 麻醉方式： GE

  診斷： Malignant tumor of rectum

  手術名稱： 1.Low anterior resection with colo-anal anastomosis2.Ileostomy3.Laparoscopy4.

  手術局部用藥： NIL

  術前預防性抗生素：
  - 抗生素名稱一： Veterin 1GM IVD由麻醉護理人員執行
  - 時間： 1240
  - 上次簽章: 陳思宇

  ，追加預防性抗生素：
  - 抗生素名稱一： Veterin 1GM IVD由麻醉護理人員執行
  - 時間： 1640
  - 上次簽章: 戴培雅
- 手術醫師： 蔣鋒帆，黃偲霖，陳映菁
- 麻醉醫師： VS 蔡志忠
-
  - 刷手護士A ： 沈佳融，時間： 1140，接替刷手一： 黃美雅，時間： 1230，接替刷手二： 陳祐媜，時間： 1500
  - 流動護士A ： 黃美雅，時間： 1140，接替流動一： 陳思宇，時間： 1200，接替流動二： 沈佳融，時間： 1300
- 更多接替及流動護士： 接替流動三： 戴培雅，時間： 1600，接替流動四： 林愛甯，時間： 1730

皮膚狀況

A-V Shunt： 無

術前： 完整

記錄者： 上次簽章: 陳思宇

術後： 不完整

部位(術後)：

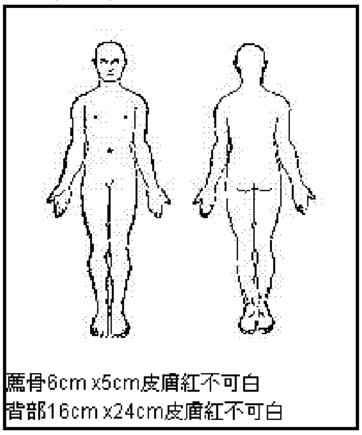

記錄者： 戴培雅

手術準備

手術臥式 單一手術臥式

-
  - 手術臥式： 截石臥式
  - 使用附件： 矽膠枕頭，腳架
  - 皮膚準備： 10%Tr.Betadine，10%Aq Betadine，Hibiscrub

- ○ 電燒： 使用電燒: 電極片位置： 右大腿，記錄者： 上次簽章: 陳思宇

止血帶T： 無

植入物

- 植入物名稱/號碼/數量/部位： ETHICON ENDO GIA 60X4.1mm X3

  EEA 29mm X1

, 記錄者： 上次簽章: 戴培雅

標本送檢

- 細菌培養 無
- 細胞學 無
- 冰凍切片 無
- 病理標本
  組織名稱一： distal cut end 1
  ,
  組織名稱二： distal cut end 2
  ,
  組織名稱三： IMA root
  , 更多組織名稱
  - ○ 組織名稱四： retum
- 截肢 無

標本交付 標本： 2 袋，標本： 0 盒，標本： 2 瓶，細胞學： 0 管，Culture Tube： 0 管，被交付醫師： R 林宜慧，

- 記錄者： 上次簽章: 戴培雅
- 時間： 1700

傷口術後敷料

傷口敷料： 傷口敷料： 無

- 請於圖上標示部位：

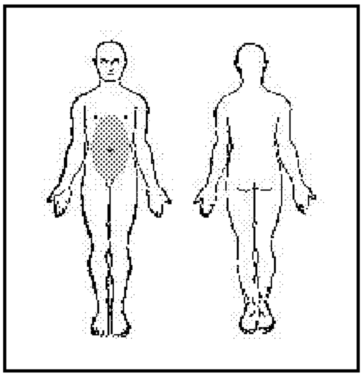

- 傷口敷料： soft cloth dresing

導管

導管	天數	導管資訊	插入日期	插入時間	更換日期	拔除日期	拔除時間	拔除原因	預計拔除/更換	預計拔除/更換日期
Epidural PCA(1)	1	部位:硬脊膜;	2020/03/26	1220						
Foley(1)	1	號碼:16 Fr.; 部位:膀胱;	2020/03/26	1230						
Penrose Drain(1)	1	號碼:12 mm.; 部位:骨盆腔;	2020/03/26	1615						
Sump NG Tube(1)	1	號碼:16 Fr.; 深度:50cm; 部位:胃;	2020/03/26	1220						
A-line (1)	1	號碼:20 Ga; 部位:左手;	2020/03/26	1230						

用物計數

- 手術中出血量： minimal c.c.
- 手術中尿量： 200 c.c.

-
  - 其它： 無

用物計數： 多切口同時縫合

- 1.器械： 正確，2.縫針： 正確，3.刀片： 正確，4.紗布： 正確，5.腹部墊： 未使用，6.紗球： 未使用，7.PEANUTS ： 未使用，8.紗條： 未使用，9.絲帶： 正確，10.針頭： 未使用，11.Vessel loop： 未使用，12.Boots： 未使用，13.Jelco： 未使用，14.棉條： 未使用，
  - 第一次：
    - 刷手護士 ： 陳祐嫥
    - 流動護士： 上次簽章: 戴培雅
    - 時間： 1615

  ,
  - 第二次：
    - 刷手護士 ： 陳祐嫥
    - 流動護士： 上次簽章: 戴培雅
    - 時間： 1630

  ,
  - 第三次：
    - 刷手護士 ： 陳祐嫥
    - 流動護士： 上次簽章: 戴培雅
    - 時間： 1700

備註

備註： crs 腹腔鏡 no. 2

- 記錄者： 上次簽章: 戴培雅
- 時間： 1700

填寫護理人員 戴培雅

正式摘要

W52 / W52

doctorRank:B
